# *Epicoccum layuense* a potential biological control agent of esca-associated fungi in grapevine

**DOI:** 10.1371/journal.pone.0213273

**Published:** 2019-03-26

**Authors:** Giovanni Del Frari, Ana Cabral, Teresa Nascimento, Ricardo Boavida Ferreira, Helena Oliveira

**Affiliations:** Linking Landscape, Environment, Agriculture and Food (LEAF), Instituto Superior de Agronomia, Universidade de Lisboa, Lisboa, Portugal; Universita degli Studi di Pisa, ITALY

## Abstract

*Epicoccum* is a genus of ascomycetes often associated with the mycobiome of grapevines (*Vitis vinifera*). *Epicoccum* spp. are found in the soil, phyllosphere, as well as in the wood, where they interact both with the plant and with other endophytes and pathogens. Wood pathogens involved in the esca disease complex, a grapevine trunk disease, are particularly concerning in viticulture, as current control strategies have proven unsatisfactory. This study investigated the interaction among *Epicoccum* spp. and three esca-associated fungi, with the aim of establishing whether they are suitable candidates for biological control.A screening conducted *in vitro*, by means of dual culture, revealed that all tested *Epicoccum* spp. inhibited the growth of pathogens *Phaeomoniella chlamydospora* and *Fomitiporia mediterranea*, while only some of them inhibited *Phaeoacremonium minimum*. *Epicoccum layuense* E24, identified as the most efficient antagonist, was tested in rooted grapevine cuttings of cultivars Cabernet Sauvignon and Touriga Nacional, under greenhouse conditions, against *P*. *chlamydospora* and *P*. *minimum*. This study revealed that the inoculation of *E*. *layuense* E24 produced a successful colonization of the wood of grapevines; in addition it did not impair the growth of the plants or induce the appearance of symptoms in leaves or in wood. Moreover, grapevines colonized by *E*. *layuense* E24 showed a considerable decrease in the wood symptomatology caused by the inoculated pathogens (by 31–82%, depending on the pathogen/grapevine cultivar), as well as a reduction in their frequency of re-isolation (60–74%).Our findings suggest that *E*. *layuense* E24 is a promising candidate for its application in biological control, due to its antagonistic interaction with some esca-associated fungal pathogens.

## 1. Introduction

Grapevine trunk diseases (GTDs) are an increasing threat to worldwide viticulture [1–3]. Affected grapevines (*Vitis vinifera* L.) exhibit lower vigor, reduced productivity and quality of yields, shorter lifespan; which, altogether, cause considerable economic losses [3,4]. The causal agents of these diseases are found in diverse groups of both ascomycetes and basidiomycetes, which can colonize the woody tissues of grapevines, interfering with the plant physiology, microbial ecology and activating plant response mechanisms [1,5]. Symptoms of an infection by trunk disease pathogens are often elusive. They are found primarily in the wood, in the form of brown streaking (longitudinal discoloration of xylem vessels), black dots, necrosis and wood decay; occasionally, symptoms may appear in other organs of the plants as well (e.g. leaves and bunches) [6,7].

One of the major GTDs is the esca disease complex which comprises five different syndromes. The first three, (i) brown wood streaking of rooted cuttings, (ii) Petri disease and (iii) grapevine leaf stripe disease (GLSD), are mainly caused by the tracheomycotic pathogens *Phaeomoniella chlamydospora* (W. Gams, Crous, M.J. Wingf. & Mugnai) Crous & W. Gams and *Phaeoacremonium minimum* (Tul. & C. Tul.) D. Gramaje, L. Mostert & Crous. These syndromes manifest in vines of different ages, the former affects newly grafted plant material, Petri disease is diagnosed in young vines, while GLDS generally affects adult plants. The fourth syndrome is (iv) esca, which is caused by different basidiomycetes, most frequently *Fomitiporia mediterranea* M. Fisch. This pathogen has the capacity to degrade lignin and induces the appearance of white rot in the wood. Lastly, (v) esca proper, that corresponds to the co-occurrence of GLSD and white rot in the same vine [8,9]. Infections occur primarily in the field, where spores of the pathogens land on fresh pruning wounds and make their way in the wood, as well as via infected propagating material [3,7,10]. Control strategies, aiming to limit the infection caused by these pathogens, have often proven unsatisfactory, offering only partial protection. Additionally, the regulatory restrictions that chemicals are facing in most countries around the world are leading researchers towards alternative measures as a priority, in which biological control is included [3,11]. Bacteria, oomycetes and fungi have been screened in order to find suitable candidates to be exploited in the biological control of esca-associated fungi. Among the tested bacteria, *Bacillus subtilis* strains have revealed promising antagonistic traits against GTD pathogens *in vitro* and *in vivo*, both in pruning wound protection and in nurseries [12–15]. On the side of oomycetes, the colonization of the root system by *Pythium oligandrum* triggered the plant defenses and reduced the extent of the wood symptomatology caused by *P*. *chlamydospora* [16]. Among fungi, tests have been conducted with *Aureobasidium* spp. [17], *Chaetomium* spp. [18], *Fusarium lateritium* [19], but the most studied species are those belonging to the genus *Trichoderma*, namely *T*. *atroviride*, *T*. *harzianum* and *T*. *longibrachiatum*, which have been used with encouraging results in pruning wound protection or throughout the different steps of grapevine propagation in nurseries [20–22]. Despite of these results, there are several other candidates that may reveal potential for biological control against esca-associated pathogens, some of which are assigned to the genera *Epicoccum*, *Cladosporium* and *Alternaria* [5,23].

Fungi belonging to the genus *Epicoccum* are ubiquitous ascomycetes (*Didymellaceae*) frequently isolated from healthy and diseased grapevine wood, being *Epicoccum nigrum* the most referred species [4,5,23]. However, there are different references to the *Epicoccum* genus, not ascribed to a species, that have been reported from healthy grapevine cuttings [24], pruning wounds [25] and mature grapevine plants [26,27]. These fungi have been thought to be endophytes or saprophytes in grapevine wood but their role on healthy or diseased wood, where they can co-inhabit with different GTDs pathogens, has not been investigated.

Several studies proved the antagonistic effect of *E*. *nigrum* against plant pathogenic agents, and it currently finds application as a biological control agent (BCA) in different crops [28–32]. The antagonistic behavior of *E*. *nigrum* is mainly attributed to the release of secondary metabolites, some of which have antifungal (e.g flavipin, epicorazine and epirodins) and anti-bacterial (e.g. beauvericin) activity [33,34]. *Epicoccum nigrum* is known as a genotypically and phenotypically highly variable species [35] and it has been hypothesized that *E*. *nigrum* could encompass different species [36]. Recent developments on the taxonomy of *Didymellaceae* fungi are contributing to clarify boundaries between genera in this family, as well as delimiting new species or combinations within the *Epicoccum* genus [37–40]. Some of these new species are represented by a restricted number of strains closely related to *E*. *nigrum* [38].

The presence of *Epicoccum* fungi in grapevines is well known, nevertheless, information regarding their interactions with the plants, their role in the microbial ecology of the wood and the interaction with GTDs pathogens is scarce. Moreover, the potential of these fungi to be exploited as BCAs against esca-associated fungi has not yet been assessed. Therefore, the aims of this study are: (i) to identify *Epicoccum* spp. currently isolated from grapevine wood in Portugal, and (ii) to gain an understanding of the type of interaction that occurs among members of the genus *Epicoccum* and esca-associated pathogens both *in vitro* and *in vivo*.

## 2. Materials and methods

### 2.1 *Epicoccum* spp. isolation

Isolates (E17, E20-E24, E27 and E28) were obtained from grapevine woody tissue sampled from canes in a vineyard, cv. Touriga Nacional, located in the Alentejo region (district of Beja), while isolate (E33) was obtained from symptomatic wood in a vineyard, cv. Touriga Nacional, located in the Lisbon region (Table 1). Wood chips, 2 mm thick, were surface disinfected by immersion in a NaClO solution (0.05% w/w active chlorine) for 1 min, followed by double-rinsing in sterile distilled water (SDW) and plated on potato dextrose agar (PDA, BD-Difco Laboratories, Detroit, MI, USA) supplemented with 250 mg l^-1^ chloramphenicol (BioChemica, AppiChem, Germany). Petri dishes were incubated at 25°C in the dark and regularly checked for the development of *Epicoccum*-like fungi, based on the cultural characteristics of colonies. The isolates were single-spored or purified by cutting off the tip of a hypha, and maintained on slants of PDA, at 5°C, until use.

**Table 1:** Isolates of *Epicoccum layuense* and related species used for phylogenetic analyses. Ex-type isolates are shown in bold type.

Species	Isolate ^1^	Host, substrate	Country	GenBank accession numbers		
				ITS	*TUB*	*RPB2*
***Didymella exigua***	**CBS 183.55**	*Rumex arifolius*	France	GU237794	GU237525	EU874850
***Epicoccum cedri***	**MFLUCC 17–1058**; KUMCC 17–0140	*Cedrus deodara* (dead land branch)	Italy	KY711170	KY711168	–
***E*. *dendrobii***	**CGMCC 3.18359**	*Dendrobium fimbriatum*	China	KY742093	KY742335	–
	LC 8146	*Dendrobium fimbriatum*	China	KY74209	KY742336	–
***E*. *italicum***	**CGMCC 3.18361**	*Acca sellowiana*	Italy	KY742099	KY742341	KY742172
	LC 8151	*Acca sellowiana*	Italy	KY742100	KY742342	KY742173
***E*. *layuense***	**CGMCC 3.18362**	*Perilla* sp.	China	KY742107	KY742349	–
	LC 8156	*Perilla* sp.	China	KY742108	KY742350	–
	E 20	*Vitis vinifera*	Portugal	MH643918	MH643928	MH643939
	E 21	*Vitis vinifera*	Portugal	MH643919	MH643929	MH643940
	E 22	*Vitis vinifera*	Portugal	MH643920	MH643930	MH643941
	E 23	*Vitis vinifera*	Portugal	MH643921	MH643931	MH643942
	E 24	*Vitis vinifera*	Portugal	MH643922	MH643932	MH643943
	E 27	*Vitis vinifera*	Portugal	MH643923	MH643933	MH643944
	E 28	*Vitis vinifera*	Portugal	MH643924	MH643934	–
	E 33	*Vitis vinifera*	Portugal	MH643925	MH643935	MH643945
***E*. *mackenziei***	MFLUCC 16–0335; KUMCC 16–0071	*Ononis spinosa* (dead aerial stem)	Italy	KX698039	KX698032	KX698035
***E*. *nigrum***	**CBS 173.73**	*Dactylis glomerata*	USA	FJ426996	FJ427107	KT389632
	CBS 125.82	Human toe nail	The Netherlands	FJ426995	FJ427106	KT389631
	LC 8158	*Poa annua*	USA	KY742111	KY742180	KY742353
	LC 5180	*Lonicera japonica*	China	KY742109	KY742178	KY742351
*E*. *nigrum* (ex-type of ***E*. *mezzettii***)	**CBS 173.38**	*Populus* (pulp of wood, in paper factory)	Italy	MH643926	MH643936	MH643946
	E17	*Vitis vinifera*	Portugal	MH643917	MH643927	MH643938
*E*. *poae*	LC 8161	*Poa annua*	USA	KY742114	KY742356	KY742183
	CGMCC 3.18363	*Poa annua*	USA	KY742113	KY742355	KY742182
***E*. *pimprinum***	**CBS 246.60**; ATCC 22237; ATCC 16652; IMI 81601	Soil	India	FJ427049	—	FJ427159

^1)^ ATCC: American Type Culture Collection, Virginia, U.S.A; CBS: Westerdijk Fungal Biodiversity Institute (formerly CBS-KNAW), Utrecht, The Netherlands; CGMCC: China General Microbiological Culture Collection, Beijing, China; IMI: International Mycological Institute, CABI-Bioscience, Egham, Bakeham Lane, U.K.; LC: Lei Cai personal collection deposited in laboratory, housed at Chinese Academy of Sciences, Beijing, China; MFLUCC: Mae Fah Luang University Culture Collection, Chiang Rai, Thailand; KUMCC: Culture collection of Kunming Institute of Botany, Chinese Academy of Sciences, Beijing, China.

### 2.2 *Epicoccum* spp. identification

The total genomic DNA of each isolate was extracted from mycelium of 10 d old cultures grown in PDA, according to Nascimento *et al*. (2001)[41]. The internal transcribed spacer regions 1 and 2 and the intervening 5.8S nrDNA region (ITS) were amplified by using the primers ITS1F [42] and ITS4 [43], the RNA polymerase II second largest subunit (*rpb2*) gene by using the primer pair RPB2-5F2 [44] and fRPB2-7cR [45] and the partial gene of β-tubulin (*tub2*) by the primers T1 and T2 [46], respectively. PCR amplifications were performed using 1× PCR buffer with 2 mM MgCl_2_ (Thermo Scientific, Lithuania), 48 μM of each dNTP, 0.32 μM of each primer, 0.5 units Taq DNA Polymerase (Dream Taq, Thermo Scientific, Lithuania) and 10–15 ng of gDNA in a final volume of 20 μL. The cycle conditions in a iCycler thermocycler (Bio-Rad, USA) were 94 °C for 5 min, followed by 40 cycles at 94 °C for 30 s, 52 °C (for ITS) or 58 °C (for *tub2*) for 30 s, and 72 °C for 1:40 min, and a final elongation at 72 °C for 10 min. For the *rpb2* gene the amplifications followed the protocol of Woudenberg *et al*. (2013)[47]. Sequencing was performed by StabVida (Portugal). Sequences generated in this study were deposited in GenBank (accession numbers MH643917 to MH643946), the alignments and trees in TreeBASE (http://purl.org/phylo/treebase/phylows/study/TB2:S23108).

Sequences of related *Epicoccum* species were retrieved from GenBank and are listed in Table 1. Alignments were performed with MAFFT version 7 ([48]; https://mafft.cbrc.jp/alignment/server/) using the L-INS-i method and manually adjusted, if necessary, in MEGA7 [49]. Maximum likelihood (ML) and Bayesian analyses were conducted for each locus and on a three-locus dataset combined using the program SequenceMatrix 1.8 [50]. ML was implemented in the CIPRES Science Gateway V 3.3 [51] using RAxML-HPC v.8 on XSEDE (8.2.9) using the GTRCAT model and 1000 rapid bootstrap inferences. Prior to Bayesian inference, the best nucleotide substitution models for each locus according to the Akaike Information Criterion were calculated in jModelTest 2.1.10 [52]. According to this software, a Hasegawa-Kishino-Yano model with proportion of invariable sites (HKY+I) was suggested for ITS dataset, a General Time-Reversible model with gamma-distributed rate (GTR+G) for *tub2* and a GTR+G+I model for *rpb2*. The Bayesian analyses were performed in MrBayes v. 3.2.6 [53]. The Markov Chain Monte Carlo sampling was set to 10 million generations, with two independent runs with four chains, one cold chain and three heated chains with a temperature value of 0.2. The tree samples of the two cold chains were compared every 1,000 generations and stopped when the average standard deviation of split frequencies fall below 0.01. Burn-in was set at 25% after which the likelihood values were stationary and the remaining trees were used to calculate posterior probabilities. Trees from different runs were then combined and summarized in a 50% majority-rule consensus tree. *Didymella exigua* (CBS 183.55) was selected as outgroup.

### 2.3 *In vitro* interactions among *Epicoccum* spp. and esca-associated fungi

*Epicoccum* spp. isolates were tested in dual culture against three esca associated-fungi, *P*. *chlamydospora* CBS 161.90 and *P*. *minimum* CBS 110713 from the CBS culture collection (Westerdijk Fungal Biodiversity Institute, Netherlands) and a local strain of *F*. *mediterranea*. All possible combinations of pathogen–*Epicoccum* spp. were tested, as well as each fungus alone or against itself. All experiments were done in 90 mm diam. Petri dishes containing 15 ml of PDA in four biological replicates. Mycelial plugs of 4 mm diam. were cut, with a cork borer, from the actively growing margin of each fungal colony and placed, facedown, 40 mm apart. Dishes were incubated at 25°C, in the dark, for 14 d, and photographed every 2 or 3 d. The size of all colonies was measured from the images generated, using Adobe Photoshop Creative Cloud (2015). The percent growth inhibition (PGI) of pathogens was calculated using the formula: PGI (%) = 100 × ((control—treatment)/control), in which the ‘control’ represents the area of each fungal colony growing against itself and ‘treatment’ the area of each colony growing against *Epicoccum* spp.

For microscope observations, a square of agar was removed from the interaction zone of the dual cultures (*E*. *layuense* isolate E24 and pathogen) and mounted on a microscope slide on a drop of 85% lactic acid (w/w) and overlaid with a cover slip. Images were captured by using a differential interference contrast microscope (Leica DM2500, Germany) equipped with a Leica DCF295 camera. Measurements of conidia were made at 1000x magnification with the Leica Application Suite (LAS) version 3.3.0 software and round to the nearest 0.5 μm. Thirty conidia of *P*. *chlamydospora* and *P*. *minimum* were measured and the means, at 95% confidence intervals, were calculated. The spore dimensions are presented as mean values with extreme values in parentheses.

### 2.4 *In vivo* interactions among *E*. *layuense* and esca-tracheomycotic fungi

**Fig 1:**
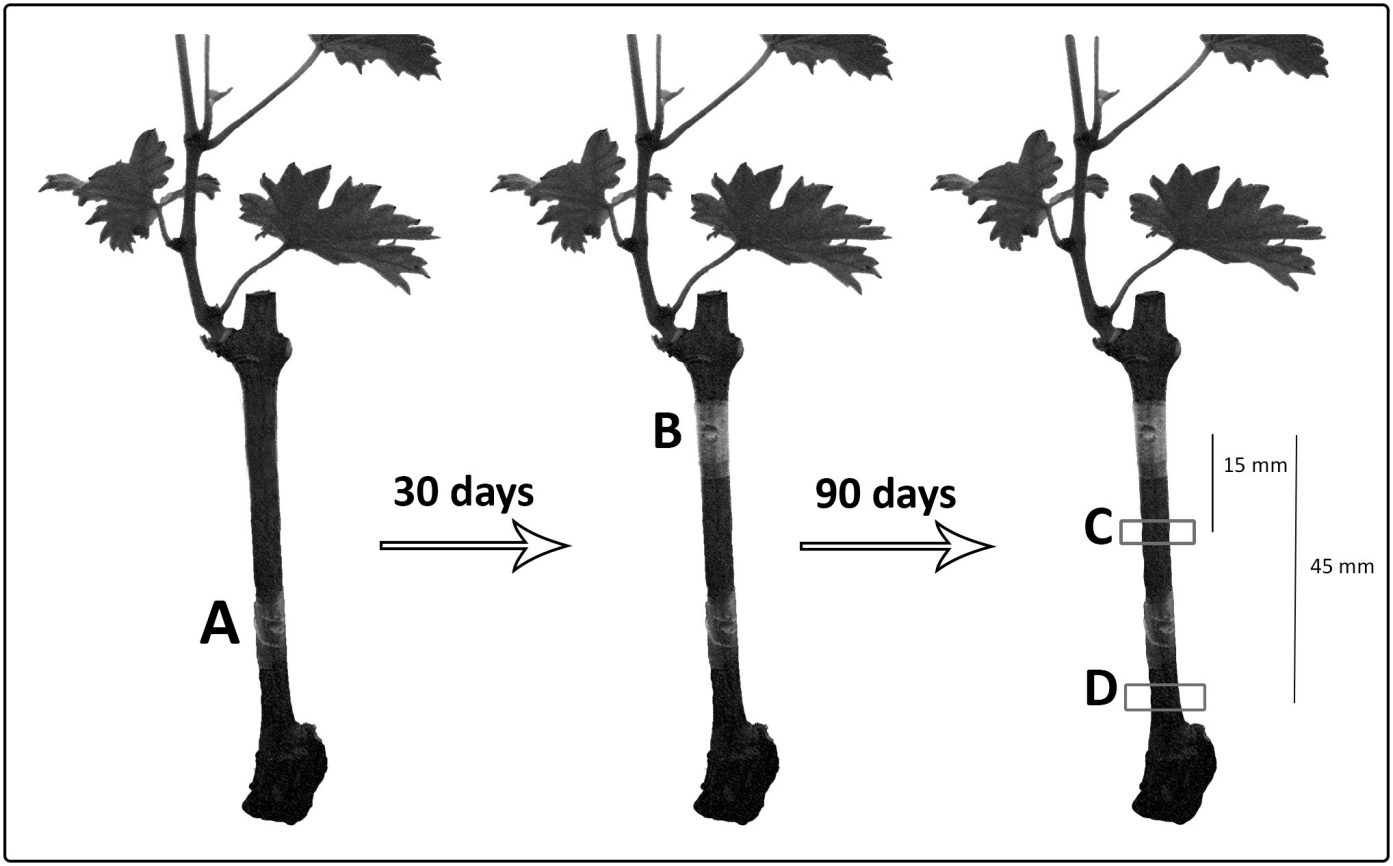
Schematic representation of the inoculation and re-isolation areas in grapevine rooted cuttings. (A) E24 inoculation via mycelium plug; (B) pathogen inoculation via spore suspension; (C) re-isolation area 15 mm below the pathogen inoculation; (D) re-isolation area 45 mm below the pathogen inoculation.

The experiments conducted *in vivo* focused on one isolate of *E*. *layuense*, namely ‘E24’, selected from the *in vitro* assays as best performing antagonist. The experiments were conducted in grapevine potted plants as follows. In a first experiment (year 2016), the isolate was tested for its pathogenicity in the wood of grapevines. One year-old canes of cv. Touriga Nacional were collected in a vineyard in the Azeitão region (Portugal), in December 2015, and left in a cold-room (4°C) for two months. Canes were divided into 3-buds-long cuttings, rooted in a warm bench, at 24°C, and potted in a mixture of peat and sand (1:1 v/v). Before inoculation, cuttings were surface disinfected with 70% ethanol. Discs of mycelium (4 mm diameter) of the isolate E24, were cut with a cork borer from the actively growing edge of a seven-days-old culture growing in PDA, and inoculated in the rooted grapevine cuttings. Mycelium discs were inserted in a wound (4 mm diameter, 4 mm deep) made in the wood with a cork borer, 40 mm below the top shoot (Fig 1A). A small piece of sterile cotton, imbibed in sterile distilled water (SDW) was placed on top of the mycelium plug and tightened to the plant with Parafilm. Mock-treated plants were inoculated with a sterile plug of PDA. Each treatment consisted of 10 biological replicates.

In a second experiment (year 2017), 3-buds-long cuttings of cvs. Touriga Nacional and Cabernet Sauvignon, rooted and potted in a mixture of peat and sand (1:1 v/v), were provided by VitiOeste nursery (Pó, Bombarral, Portugal). The following fungal combinations were tested: (i) E24 alone, (ii) *P*. *chlamydospora* CBS 161.90 and (iii) *P*. *minimum* CBS 110713 alone, (iv, v) combination E24 –each pathogen, and (vi) mock-control; with each combination consisting of 10 biological replicates.

All inoculations with the isolate E24, as well as the mock-control, were carried out as described in the first experiment (mycelial discs). The inoculation of the pathogens occurred one month later, a 50 μl aliquot of a suspension containing approximately 2000 conidia was deposited in a fresh wound (4 mm diameter, 4 mm deep) (Fig 1B) and covered with Parafilm. Mock-treated controls were inoculated with 50 μl of SDW. The conidial suspension was prepared by flooding 14 days-old PDA cultures of each pathogen with SDW, and the conidia were dislodged from the mycelium with a sterile glass rod. The suspension was filtered through a double layer of cheesecloth, the conidial concentration was determined using a hemocytometer and adjusted to 1 ×10^5^ conidia/ml with SDW.

After inoculation, plants were randomly placed in a greenhouse equipped with fan and pad evaporative system (24±5°C day/18°C night) and watered three times a week, or when needed. Fortnightly treatments with meptyldinocap (35.7% p/p) or sulfur wettable powder (80% p/p) were carried out to prevent powdery mildew (*Erysiphe necator*) infections.

Three months after the pathogens inoculation, plants were visually inspected for the appearance of foliar symptoms and the length of the shoots was recorded. Afterwards, vines were uprooted, their bark removed and longitudinal sections were made in order to assess the presence and extent of symptoms in the wood (brown streaking), departing from the inoculation points.

Pieces of wood (approx. 4 mm^3^) were cut transversally 30 mm above the area A for the first experiment, and areas C and D (Fig 1) for the second, to undergo re-isolation. The re-isolation of the pathogens occurred as follows. Four pieces of wood were taken from each level (C and D) of each plant, they were surface disinfected by flame, followed by immersion for 1 min in a NaClO solution (0.35% w/w as active chlorine), double rinsed in SDW (1 min each), dried with sterile filter paper and plated on PDA amended with 250 mg l^-1^ chloramphenicol. Petri dishes were incubated at 25°C, in the dark, for 21 d. The re-isolation of E24 was achieved as described for its isolation. The percentage of re-isolation was calculated as the proportion of wood pieces from which fungal colonies were recovered over the total number of pieces of wood plated for each plant.

### 2.5 Data analysis

All data were subjected to analysis of variance (ANOVA), and statistically significant means were compared using the Tukey *post hoc* test at a 5% significance level (STATISTICA 8.0). Before analysis, arcsine-square root transformation was performed for data expressed as percentage. For each experiment, the following analyses were carried out: (i) one-way ANOVA model to evaluate the *in vitro* effect of *Epicoccum* spp. on esca-associated fungi; (ii) two-way ANOVA model to assess the effects of the grapevine cultivar, fungal inoculation (E24, *P*. *minimum* and *P*. *chlamydospora*), and their interaction, upon the shoot length and brown wood streaking length; (iii) three-way ANOVA model to assess fungal re-isolation in which, in addition to the grapevine cultivar and fungal inoculation variables, two regions of fungal re-isolation were considered (Fig 1C and 1D), as well as their interactions. Data recorded from mock-treated plants were used to estimate any infection of rooted grapevine cuttings before inoculation and, therefore, they were not included in the statistical analyses.

## 3. Results

### 3.1 *Epicoccum* spp. identification

**Fig 2:**
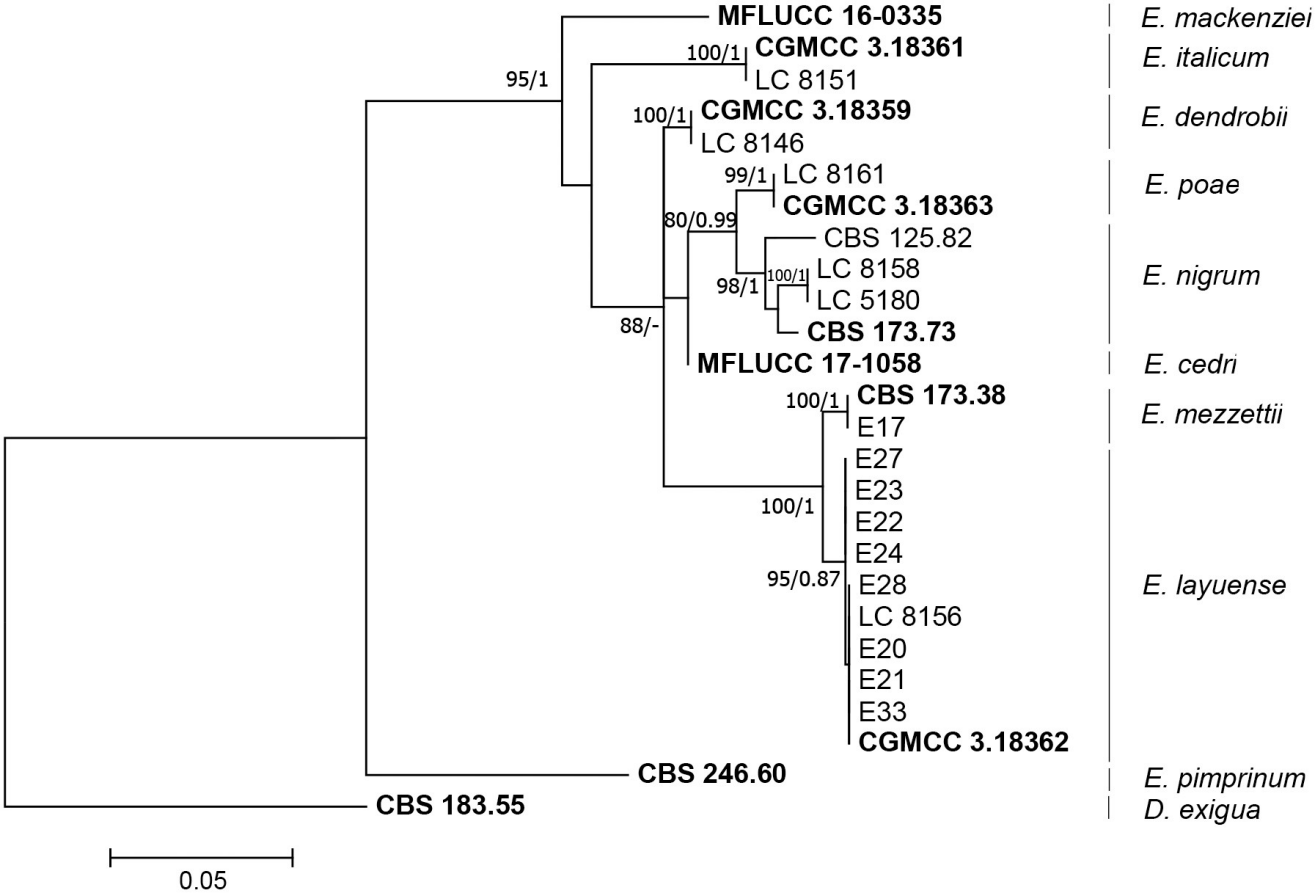
Phylogenetic tree inferred from a Maximum likelihood analysis based on a concatenated alignment of ITS, *rpb2* and *tub2* sequences of 25 isolates of *Epicoccum* and one isolate of *Didymella*. The RAxML bootstrap support values (MLBS) and Bayesian posterior probabilities (BPP) are given at the nodes (BPP/MLBS). The tree was rooted to *Didymella exigua (*CBS 183.55). Ex-type cultures are emphasised in bold type. The scale bar indicates 0.1 expected changes per site.

The three locus alignment contains 25 ingroup isolates and one outgroup *Didymella exigua* (CBS 183.55) and a total of 1422 characters including alignment gaps (490 for ITS, 596 for *rpb2* and 336 for *tub2*), from which 1154 were conserved and 154 were parsimony-informative (12 for ITS, 111 for *rpb2* and 31 for *tub2*). The tree topologies obtained by ML and Bayesian analysis of individual loci and for the combined alignments were essentially congruent, therefore, only the ML tree is shown, with bootstrap support values (MLBS) and Bayesian posterior probabilities (BPP) indicated at the nodes (Fig 2). The ITS loci were the least informative resolving only four out of nine taxa present in the dataset, whereas the other two loci, *rpb2* and *tub2*, were able to resolve all the taxa.

Among the nine isolates of *Epicoccum* obtained from grapevine, eight of them (E20-E24, E27, E28 and E30) cluster within the *E*. *layuense* clade, and one (E17) clusters with the ex-type strain of *E*. *mezzettii* (CBS 173.38), whose current name is *E*. *nigrum*. From here on, the isolate E17 will be referred as *E*. *mezzettii*, in order to distinguish it from the others belonging to *E*. *nigrum* species.

### 3.2 *In vitro* interactions among *Epicoccum* spp. and esca-associated fungi

**Fig 3:**
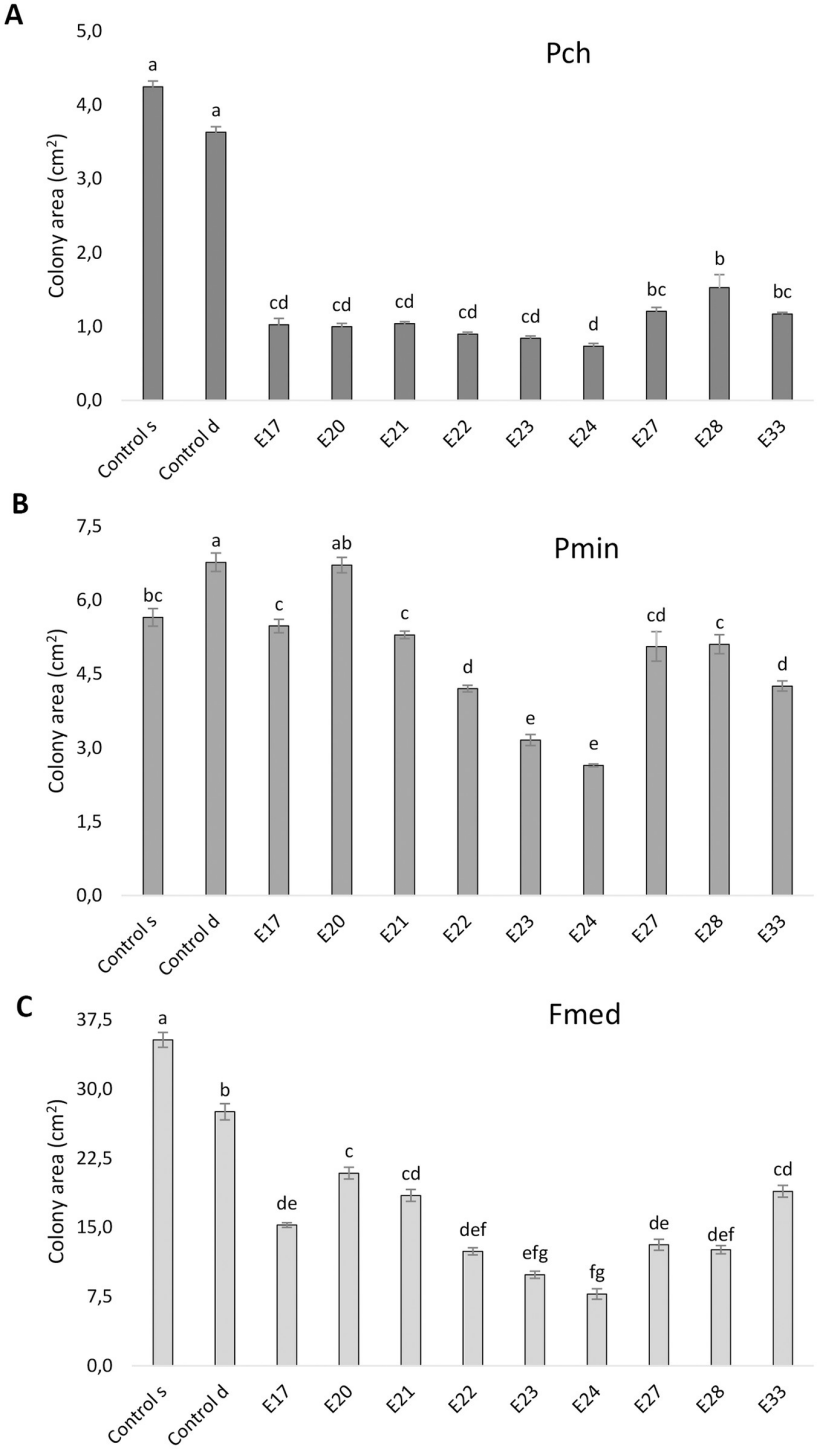
*In vitro* inhibition of esca-related fungi by *Epicoccum* spp. isolates. Growth was measured 14 days post-inoculation on PDA medium in dual-culture with (A) Pch, *Phaeomoniella chlamydspora*; (B) Pmin, *Phaeoacremonium minimum* and (C) Fmed, *Fomitiporia mediterranea*. ‘Control s’ and ‘Control d’ indicate the colony area of the pathogen in single culture or dual culture, respectively, and ‘E’ indicates the *Epicoccum* isolate. Error bars represent the standard deviation from the mean; different letters indicate statistically significant differences (Tukey *post hoc* test, p ≤ 0.05).

*Epicoccum mezzettii* (E17) and all the strains of *E*. *layuense* inhibited the growth of *P*. *chlamydospora* and *F*. *mediterranea*, after 14 d of growth on PDA medium, by the dual-culture method, while only some strains of *E*. *layuense* (E22-E24 and E33) were able to inhibit *P*. *minimum* (Fig 3).

**Fig 4:**
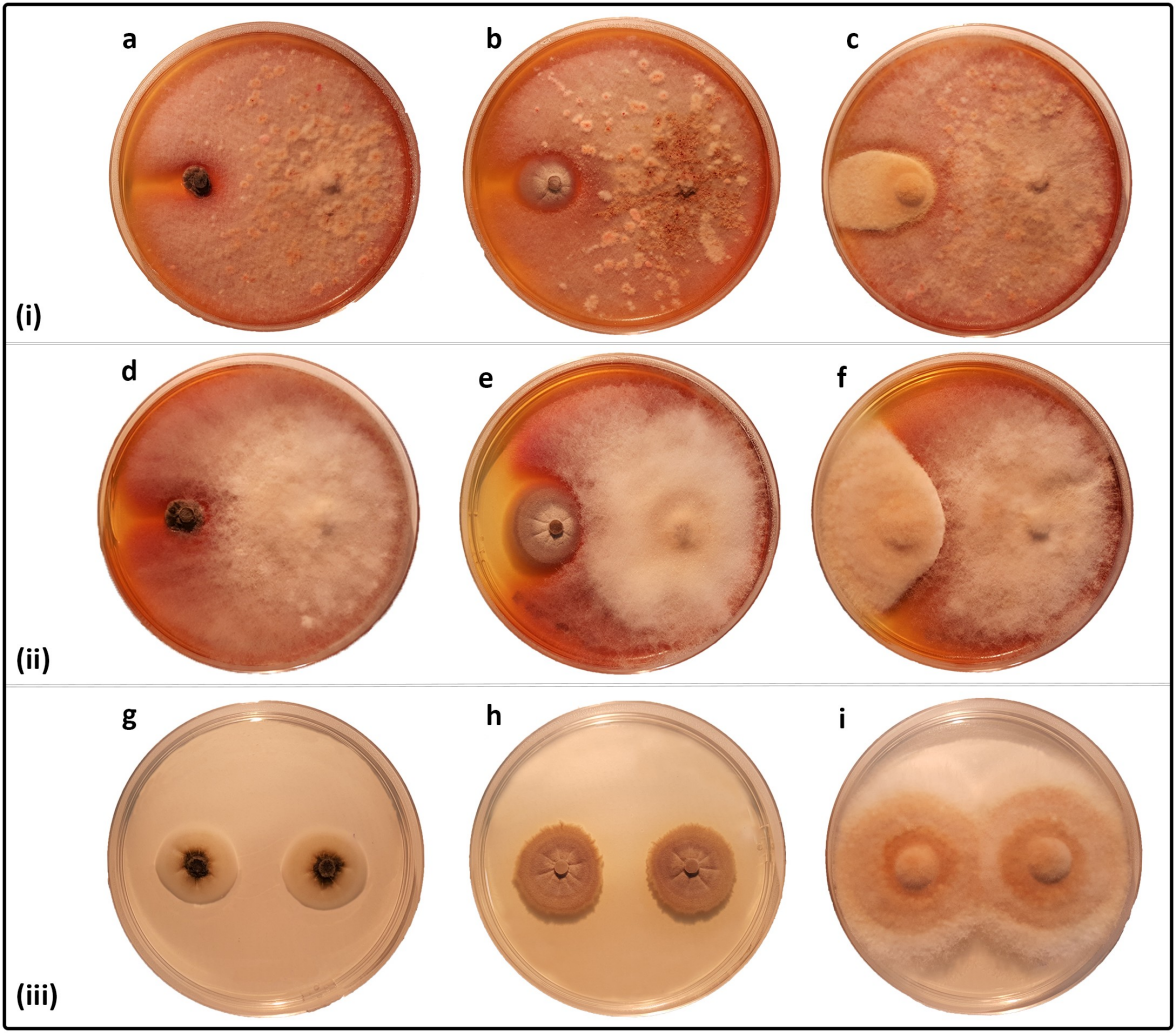
Interaction pathogen—Antagonist or pathogen—Pathogen after 14 d of incubation on PDA. (i) *Phaeomoniella chlamydospora* (a), *Phaeoacremonium minimum* (b) and *Fomitiporia mediterranea* (c) inoculated on the left side, against *Epicoccum layuense* strain E24 inoculated on the right; (ii) the same pathogens against *E*. *mezzettii* strain E17, (iii) dual cultures of each pathogen.

The growth behavior of the confronting fungi varied depending on the different fungal combinations. *Epicoccum mezzettii* (E17) was the only fungus capable of clearly overgrowing all three pathogens (Fig 4(ii)), while the remaining isolates (E20-E24, E27, E28 and E33), all assigned to *E*. *layuense*, were able to inhibit the pathogens without a physical colony contact, when observed by the naked eye.

The interaction *Epicoccum—P*. *chlamydospora* was characterized by a great reduction of the colony size of the pathogen, when compared to both single and dual culture controls (Fig 3A). The average reduction was of 71.0%, with the best performer being E24 that reduced the growth of *P*. *chlamydospora* by 79.9% (Table 2), as exemplified in Fig 4(a), 4(d) and 4(g). A time-lapse picture sequence is presented in the supporting information (S1 Fig).

**Table 2:** Percent growth inhibition of esca-associated fungi by *Epicoccum* spp.

*Epicoccum* spp.	Isolate	PGI (%)^1^		
		*P*. *chlamydospora*	*P*. *minimum*	*F*. *mediterranea*
***E*. *mezzettii***	E17	71.8	19.1	44.6
***E*. *layuense***	E20	72.6	0.9	24.2
	E21	71.4	21.8	32.9
	E22	75.3	37.9	55.0
	E23	76.9	53.4	64.2
	E24	79.9	61.0	71.8
	E27	66.8	25.3	52.4
	E28	58.0	24.6	54.3
	E33	67.8	37.2	31.4

^1^Calculated using the formula: PGI (%) = 100 × ((control—treatment)/control) in which the control represents the area of each fungal colony growing against itself and treatment the area of each colony growing against *Epicoccum* spp.

**Fig 5:**
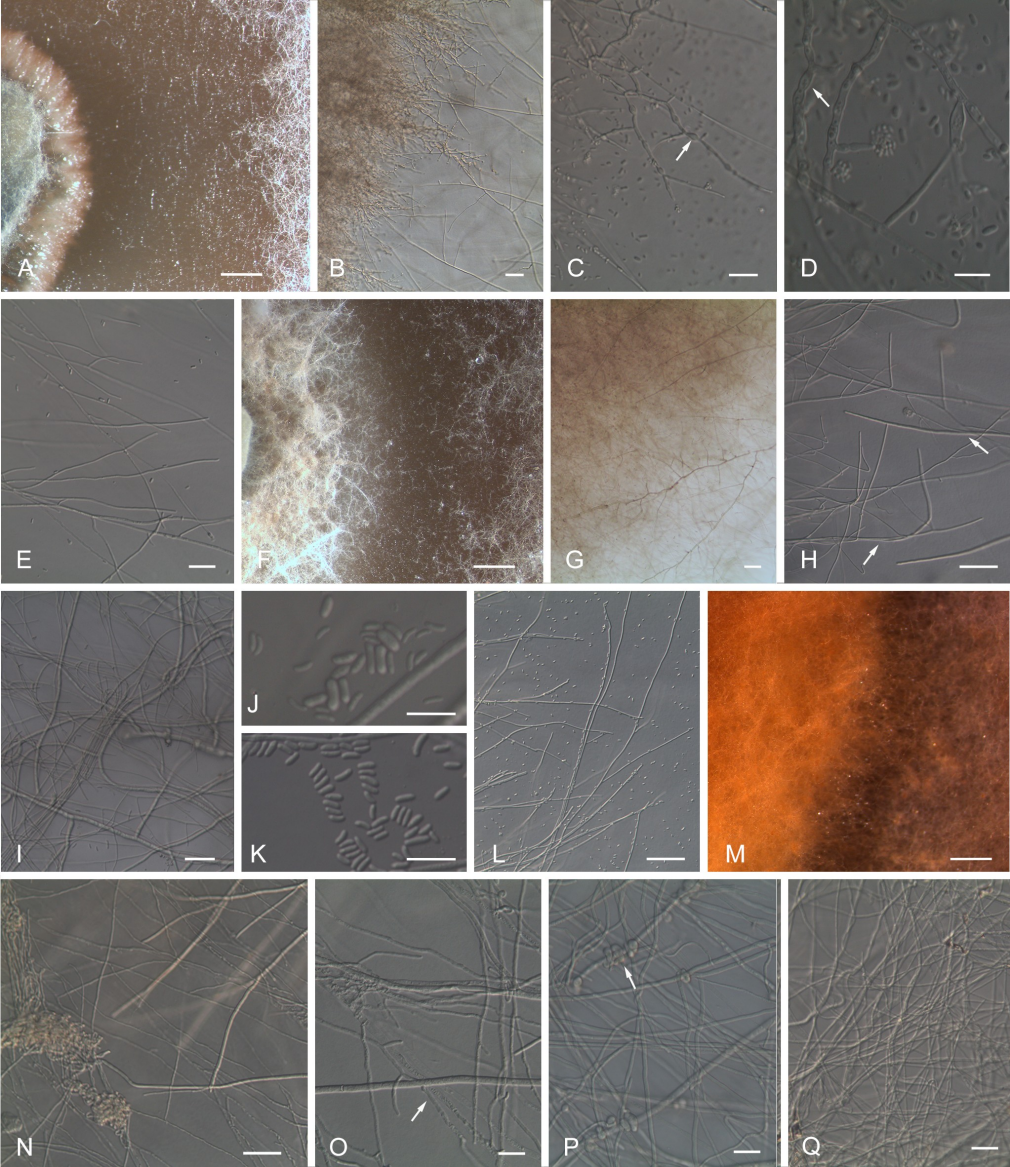
Morphological changes of vegetative structures of esca-associated fungi upon interaction with *Epicoccum layuense* isolate E24 in dual culture plates. *Phaeomoniella chlamydospora* isolate CBS 161.90 (left side) and *E*. *layuense* (right side) at interaction area at day eight (A); hyphae of *P*. *chlamydospora* with swollen chlamydospores (arrow) and an increasing of sporulation in the contact line with *E*. *layuense* (B-D) when compared to *P*. *chlamydospora* growing alone (E) at day ten. *Phaeoacremonium minimum* isolate CBS 110713 (left side) and *E*. *layuense* (right side) at the interaction zone at day eight (F); intermingled hyphae of both fungi at day ten (G) after the hyphae of *P*. *minimum* have tried to change their growth direction (left arrow) to avoid the contact with the antagonist (right arrow) at day six (H); agglomerates of *P*. *minimum* hyphae intermingled with the antagonist hyphae (I), conidia of *P*. *minimum* from the interaction zone (J) when compared to conidia from the *P*. *minimum* single culture (K), and hyphae of *P*. *minimum* growing alone (L), all at day ten. *Fomitiporia mediterranea* (left side) and *E*. *layuense* (right side) at the interaction zone at day eight (M); agglomerates and strands of *F*. *mediterranea* hyphae in an attempt to block the advance of *E*. *layuense* (N); hyphae of *F*. *mediterranea* denoting plasmolysis (arrow) (O) and clamp connections (arrow) (P); mycelium of *F*. *mediterranea* growing alone (Q). Scale bars represent A, F, M = 1 mm; B, G, H, L, N = 50 μm; C, E, I, O-Q = 20 μm; D, J, K = 10 μm.

Microscopic observations of the interaction between *E*. *layuense* (E24) and *P*. *chlamydospora* (CBS 161.90) revealed that the growth of *P*. *chlamydospora* ceases before the contact with the antagonist. When the hyphae of *E*. *layuense* are within 3–4 mm distance of *P*. *chlamydospora* hyphae, the growth slackens and the hyphae become sparser, growing below the agar surface without visible aerial mycelium (Fig 5A). The *E*. *layuense* isolate extends its growth towards *P*. *chlamydospora*, surrounding it, with an intermingling of the hyphae but without overlapping the pathogen (Fig 5D). The *P*. *chlamydospora* isolate reacts to the presence of the antagonist increasing the conidia production along the contact line and the hyphae differentiate swollen cells that remind chlamydospores (Fig 5E and 5F). No differences were observed between the conidia dimensions of *P*. *chlamydospora* withdrawn from the interaction zone and the conidia of this pathogen growing alone.

All antagonists but one (E20) significantly inhibited the growth of *P*. *minimum* when compared to the dual culture control (Fig 3B). The single culture control grew significantly less than the dual, which suggests a possible growth-promoting interaction between *P*. *minimum* colonies. Also in this case, the interaction between E24 and *P*. *minimum* caused the greatest reduction in the colony size of the pathogen, amounting for a 61.0% decrease (Table 2; Fig 3B). A time-lapse picture sequence is presented in the supporting information (S2 Fig).

Microscopic details of the interaction *E*. *layuense* (E24) and *P*. *minimum* (CBS 110713) reveal that both fungi slacken their growth when they approach each other. The pathogen, sensing the presence of *E*. *layuense*, alters the growth direction of their hyphae, in an attempt to avoid contact (Fig 5H), although at a later stage the intermingling and overlapping of hyphae of both fungi become visible (Fig 5G and 5I). The conidia of *P*. *minimum* withdrawn from the interaction zone are larger and narrower ((3.5–)4.5–5.0–5.5(–6.5) × (1.0–)1.0–1.3–1.5(–1.5) μm) than those collected from the single culture of *P*. *minimum* ((3.0–)3.5–4.1–4.5(–5.5)×(1.0)–1.5–1.5–1.5(–2.0) μm), having a greater length-width ratio ((3.0–)3.5–4.1–4.5(–6.0)) when compared to the control ((1.5–)2.5–2.7–3.0(–3.5)).

Regarding *F*. *mediterranea*, *E*. *layuense* (E24) proved to be one of the most efficient inhibitor, inducing a colony size reduction of 71.8% (Table 2; Fig 3C). All other *Epicoccum* isolates were effective in inhibiting the mycelial growth of this pathogen, although there were significant differences among them (Fig 3C). A time-lapse picture sequence is presented in the supporting information (S3 Fig).

Microscopic details of the confronting zone between *E*. *layuense* (E24) and *F*. *mediterranea* reveal that after six days of incubation the hyphae of both fungi come into contact and the pathogen responds by entangling their hyphae, forming hyphal strands. In addition, degradation of the hyphal tips and formation of clamp connections are observed. On the bottom of the dual culture it is visible a brown streak along the line of contact of fungi corresponding to a multiplication of branches piles up the hyphae to prevent the contact with the antagonist (Fig 5M–5Q).

### 3.3 *In vivo* interactions between *E*. *layuense* and esca-tracheomycotic fungi

**Fig 6:**
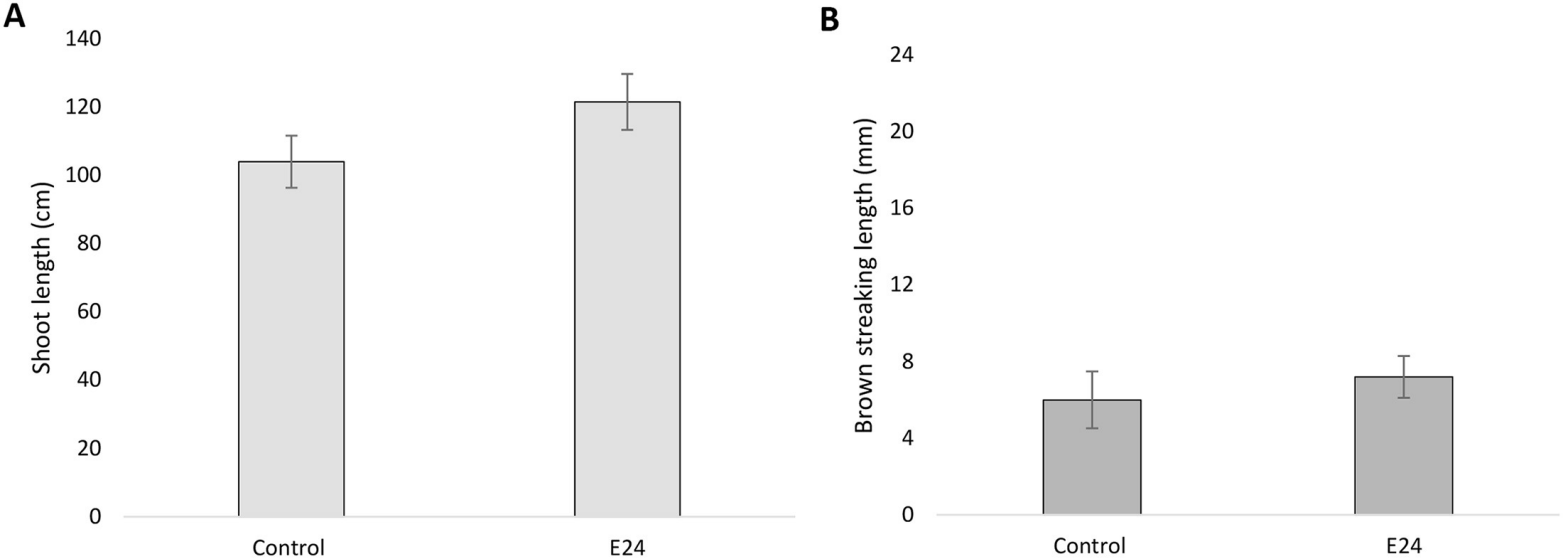
Effects of *Epicoccum layuense* E24 on grapevines. Measurements of the shoot length (A) and brown wood streaking length (B), in cv. Touriga Nacional, four months after inoculation. Error bars represent the standard deviation from the means.

The first experiment was carried out to infer whether E24 isolate could induce symptoms of a wood disease, in grapevine potted plants of cv. Touriga Nacional. This investigation revealed that, after four months of incubation, inoculated plants did not present any foliar symptoms, such as chlorosis, spotting or tiger stripes pattern, and the shoot length of inoculated plants was not significantly different from the control (Fig 6A). The examination of the stem for the presence of wood symptoms, in longitudinal sections, revealed no relevant brown-wood streaking, when compared to the mock-treated plants. The discoloration observed in woody tissue was short in length and related to the wound produced in the inoculation point, being present both in inoculated and mock-treated vines (Fig 6B). By the end of the assay, the E24 isolate was consistently yielded from inoculated plants, by means of *in vitro* re-isolation, and never from the control, thus indicating its potential for the inner colonization of grapevine.

The results from the first experiment, revealing that *E*. *layuense* E24 was unable to produce wood symptoms and affect the grapevine growth, led to a second experiment, performed in 2017, in which two grapevine cultivars were evaluated (Touriga Nacional and Cabernet Sauvignon). In this case, the *in vitro* re-isolations of the inoculated fungi aimed to assess the presence of both pathogens and antagonist in all combinations, and the re-isolations occurred both 15 mm and 45 mm below the point where the pathogens were inoculated (Fig 1).

Results revealed that the artificial inoculation of *E*. *layuense* (E24) did not produce external symptomatology on both grapevine cultivars, four months after inoculation. The pathogens, *P*. *chlamydospora* and *P*. *minimum*, whose inoculation was performed one month after that of the antagonist, were also unable to induce external symptomatology.

The growth of the green shoots, and therefore their final length, was not significantly influenced by the presence of either pathogens and/or antagonist, in both cultivars (p >0.05; Table 3).

**Table 3:** Shoot length (cm) of grapevine potted plants inoculated with water (control) or with *Epicoccum layuense* strain E24, *Phaeoacremonium minimum* (Pmin), *Phaeomoniella chlamydospora* (Pch) and the combination of E24 with Pmin (E24 x Pmin) or Pch (E24 x Pch).

Grapevine cultivar	Water control	E24	Pmin	Pch	E24 x Pmin	E24 x Pch
**Touriga Nacional**	77.2 ± 12.1	61.4 ± 12.2	62.7 ± 13.6	75.0 ± 13.7	63.6 ± 16.8	60.3 ± 10.9
**Cabernet Sauvignon**	89.2 ± 12.2	81.4 ± 19.7	70.6 ± 13.6	79.1 ± 14.0	82.9 ± 18.6	71.7 ± 10.6

Measurements were taken three months after the inoculation of the pathogens.

**Fig 7:**
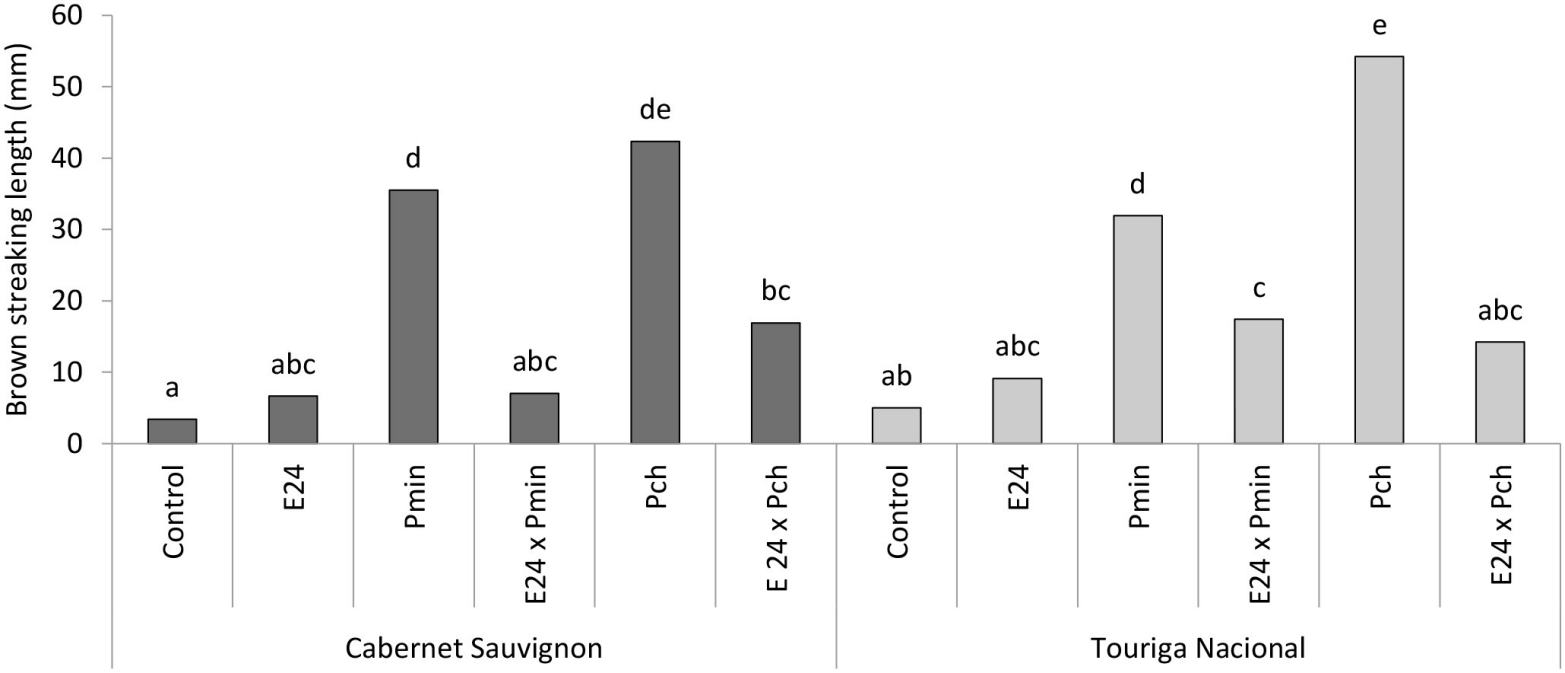
Brown wood streaking length in inoculated grapevine potted plants. Grapevines were inoculated with water (control) or with *Epicoccum layuense* strain E24, *Phaeoacremonium minimum* (Pmin) and *Phaeomoniella chlamydospora* (Pch) alone, or combined with E24 (E24 x Pmin and E24 x Pch). Bars followed by the same letter do not differ statistically according to the Tukey’s test (p = 0.05).

While the inoculation of E24, *P*. *minimum* or *P*. *chlamydospora* did not produce any external symptom on grapevines of both cultivars, the two pathogens induced the appearance of internal symptoms of brown wood streaking that differed significantly in length from those inoculated with E24 isolate and mock-treated plants (Fig 7). In both cultivars, the wood symptoms due to E24 inoculation alone were not significantly greater than the controls, which support results from the previous experiment (first year) on the behavior of *E*. *layuense* on grapevine wood. In Touriga Nacional, the wood streaking significantly decreased in length due to the E24—pathogen interaction, for both pathogens. The same behavior was recorded in Cabernet Sauvignon for both interactions, namely E24 x *P*. *minimum* and E24 x *P*. *chlamydospora*. In both cultivars, the inoculation of E24 reduced or completely prevented the negative effects of the pathogens, in regards to the development of brown wood streaks.

The presence of the inoculated fungi, calculated as the frequency of re-isolation, supported the insights gained by the brown wood streaking measurements. A more comprehensive understanding of the pathogen—antagonist interaction was obtained thanks to the two re-isolations areas examined, 15 mm and 45 mm below the pathogens’ inoculation point (Fig 1C, 1D and 1B). The presence of *E*. *layuense* E24 was also assessed in the same areas as for the pathogens.

The frequency of re-isolation of E24 in inoculated plants revealed a significant interaction among ‘grapevine cultivar’, ‘re-isolation area’ and ‘fungal inoculation’ (p < 0.05), in which the ‘grapevine cultivar’ and the ‘fungal inoculation’ were responsible for the major effects (p ≤ 0.001), while no meaningful differences emerged from the variable ‘re-isolation area’. The average frequency of re-isolation of E24 in control plants of Cabernet Sauvignon was 68.8% and a similar value was recorded in Touriga Nacional (58.8%), highlighting a successful colonization of the woody tissues in the areas near the inoculation points, thus revealing that E24 could colonize indifferently the wood tissues above and below its inoculation point.

**Fig 8:**
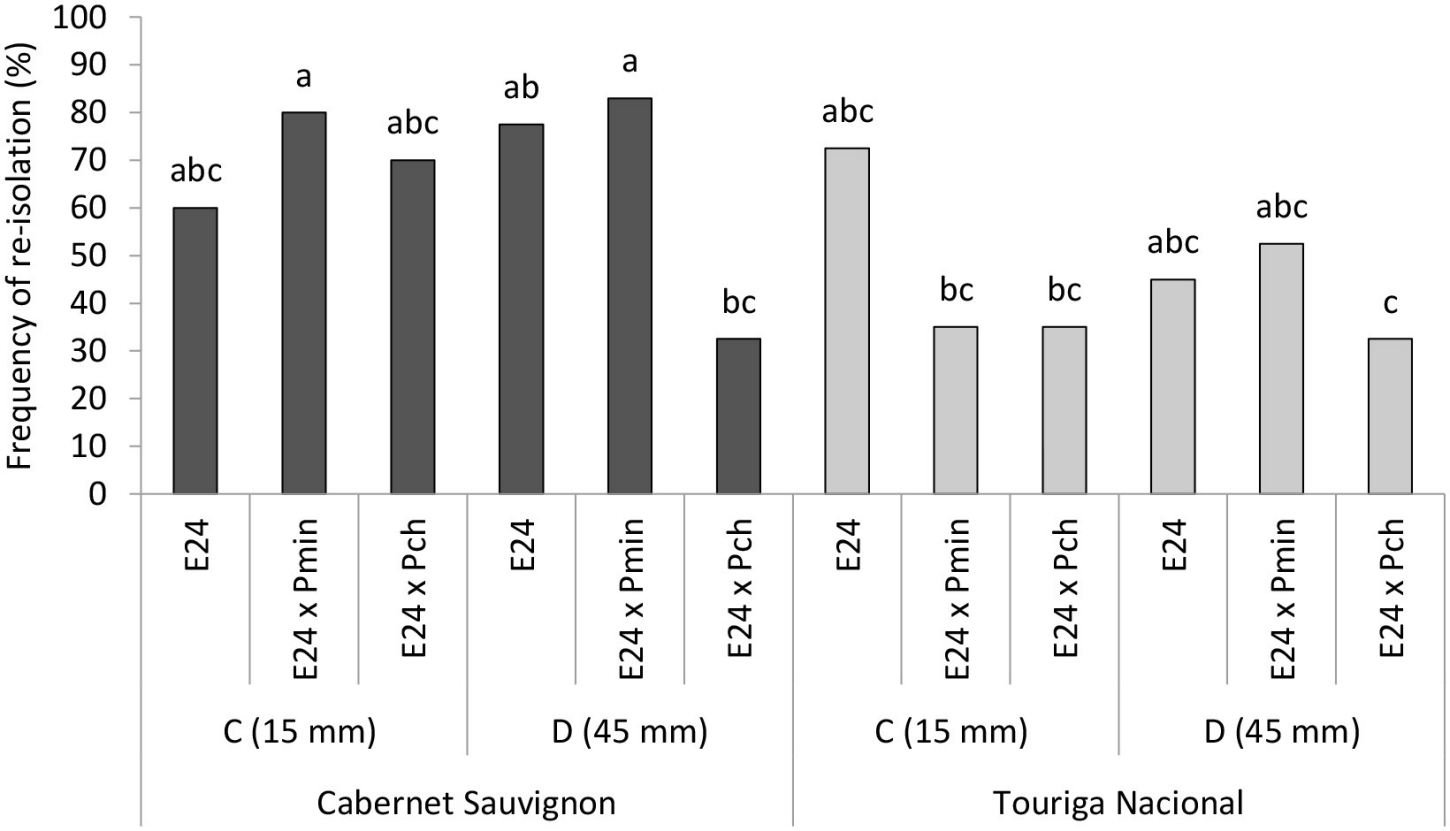
Frequency of *Epicoccum layuense* re-isolation from two grapevine cultivars and two areas of re-isolation (C 15 mm and D 45 mm). *E*. *layuense* was inoculated alone (E24) or in combination with *Phaeoacremonium minimum* (E24 x Pmin) or *Phaeomoniella chlamydospora* (E24 x Pch). Different letters on the top of the bars represent statistical differences according to the Tukey’s test (p = 0.05).

Although some variation has been observed when *Epicoccum* E24 re-isolation means are compared, neither *P*. *minimum* nor *P*. *chlamydospora* have significant detrimental effect on the antagonist re-isolation (Fig 8).

**Fig 9:**
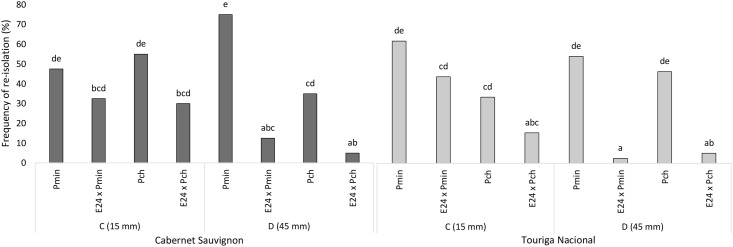
Frequency of re-isolation of inoculated pathogenic fungi. *Phaeoacremonium minimum* (Pmin) and *Phaeomoniella chlamydospora* (Pch) were either inoculated alone, or in combination with *Epicoccum layuense* E24 (E24 x Pmin, E24 x Pch). Re-isolations occurred at 15 mm (C) and 45 mm (D) below the pathogens’ inoculation point in cultivars Cabernet Sauvignon and Touriga Nacional. Different letters indicate statistically significant differences after a Tukey’s *post hoc* test (p = 0.05).

The frequency of re-isolation of pathogens, when inoculated in absence or presence of *E*. *layuense* E24, is presented in Fig 9. The three-way ANOVA revealed a significant interaction among ‘grapevine cultivar’, ‘re-isolation area’ and ‘fungal inoculation’ (p = 0.0029), with positive effects coming from the ‘re-isolation area’ (p = 0.0017) and ‘fungal inoculation’ (p < 0.001).

By examining the ‘area of re-isolation’ C (15 mm), it is possible to infer that, for both pathogens and cultivars, the frequency of re-isolation of *P*. *minimum* and *P*. *chlamydospora* is lower in the presence of E24, although never significantly different from the positive controls (inoculated with the pathogen alone). In contrast, in ‘area of re-isolation’ D (45 mm) there is a strong decrease in the presence of both pathogens in both grapevine cultivars. The re-isolation of *P*. *minimum*, when co-inoculated with E24, was reduced by 83.3% in Cabernet Sauvignon and 95.2% in Touriga Nacional. A similar trend was registered for *P*. *chlamydospora*, where the decrease in the frequency of re-isolation was of 85.7% in Cabernet Sauvignon and 88.9% in Touriga Nacional.

## 4. Discussion

### 4.1 *Epicoccum* spp. identification

In this study, a collection of *Epicoccum* spp. obtained from wood of grapevine plants in Portugal was identified morphologically and by gene sequencing (ITS, *rpb2* and *tub2*). Results revealed two *Epicoccum* species within the collection, *E*. *layuense* and *E*. *mezzettii*, being *E*. *layuense* the most represented. This species is phylogenetically closely related to *E*. *nigrum* and *E*. *poae* and it was recently reported from leaves of *Perilla* sp. in Tibet, China [38]. In turn, *E*. *mezzettii*, represented by the type strain CBS 173.38 and E17, although considered synonym of *E*. *nigrum* (Mycobank), proved to be a different species.

*E*. *nigrum*, apparently not represented in our collection, is the most reported species of the genus, in grapevine wood. However, *E*. *nigrum* was for long considered a single variable species, encompassing high diversity [36], and it is possible that other closely related species (e.g. *E*. *layuense* or *E*. *mezzettii*) have been misidentified as *E*. *nigrum*, before the taxonomic reassessment of the genera within *Didymellaceae* [38,54].

### 4.2 *Epicoccum layuense* E24 and grapevines

Strains of *E*. *nigrum* are known as potential biological control agents (BCAs) in grapevine, against fungal aerial diseases, as well as in other pathosystems [29,30,55–57], but their role as a grapevine wood endophyte, along with that of other closely related species, has not been addressed. This study reveals the inability of *E*. *layuense* E24 to impair the growth of grapevines and to induce leaf and wood symptoms on inoculated plants of cvs Touriga Nacional and Cabernet Sauvignon, within the timings of the experiments. These results support the current understanding that *Epicoccum* spp. are not associated with any grapevine disease. Long-term studies are necessary to confirm this conclusion, as wood pathogens grow slowly and plants may remain externally asymptomatic for a number of years after an infection has taken place [58].

Despite *Epicoccum* spp. are often associated with the endosphere, phyllosphere and rhizosphere of grapevine plants [23,59,60], to date, it is not clear the mode of colonization of the wood. Our results show that the inoculation of a mycelium plug of *E*. *layuense* produces a successful colonization in one-year-old cuttings of both tested cultivars, also revealing that its antagonist behavior was not influenced by grapevine cultivar.

### 4.3 *In vitro* interaction between *Epicoccum* spp. and esca-associated fungi

The various syndromes that constitute the esca disease complex are an increasing concern in viticulture for their unrestrained spread, which highlights the need to apply immediate and effective control strategies to prevent what may become the 21^st^ century phylloxera [61]. Success stories of the application of BCA against grapevine diseases are many [62], and their use could prevent several of the side effects that result from the application of fungicides [63]. Grapevine wood is characterized by a complex microbiome [4,23,64] comprising hundreds of species that interact with one another, and the role that some of these organisms play in preventing infections or slowing the spread of grapevine trunk pathogens must be further investigated. In this work, we present an overview of the interactions that lie between *Epicoccum* spp. and three esca-related fungi.

Although *E*. *layuense* has never been studied as an antagonist, the taxonomic proximity of this species to *E*. *nigrum* suggests that it may act in a similar way. Several studies demonstrated antibiosis among *E*. *nigrum* and different plant pathogenic fungi, including hyperparasitism [31]. The interaction types may vary, ranging from a pure chemical one [30] to a combination of chemical and physical [33,56].

Our work shows a species-specific interaction of *Epicoccum* spp. over *P*. *chlamydospora*, *P*. *minimum* and *F*. *mediterranea*, where *E*. *mezzettii* overgrows all three pathogens, while for *E*. *layuense* there was mutual inhibition. The fast growth of *E*. *mezzettii* and *E*. *layuense*, along with the diffusion in the culture medium of pigments, suggest that the antagonism is primarily due to competition for space, nutrients and probably chemical interaction. These claims are also supported by previous reports of *E*. *nigrum—*pathogen interactions [33,55,65]. The different pathogens behavior reported in response to the presence of *E*. *layuense* E24, such as (i) the increase in the conidia production along the contact line in *P*. *chlamydospora*, (ii) the alteration of the growth direction of the hyphae in *P*. *minimum* and (iii) the formation of hyphal strands, degradation of the hyphal tips and formation of clamp connections in *F*. *mediterranea*, are considered antagonistic interactions in other studies where different fungi were confronting [66–68].

However, the in-depth study of these interactions, as well as the chemical nature of the pigments produced by *E*. *layuense* E24 and by *E*. *mezzettii*, was not covered in this study and needs to be further investigated.

### 4.4 *In vivo* interaction between *Epicoccum layuense* E24 and esca-tracheomycotic fungi

The *in vivo* study focused on the interaction between *E*. *layuense* E24 and the two tracheomycotic fungi *P*. *chlamydospora* and *P*. *minimum*. These pathogens are the most frequently isolated from symptomatic young grapevines and they are responsible for the brown wood streaking appearance, which is a parameter of our interest. Therefore, in this part of the study, *F*. *mediterranea* was not further examined.

In the greenhouse experiment, the inoculation of both pathogens did not induce any significant external symptom, most likely because of the relatively short amount of time that the pathogens had to interact with the plant. Nevertheless, the evident brown streaking, that manifested from the inoculation point, revealed that a pathogenic mechanism had taken place in the wood, and the role of the pathogens in its occurrence was confirmed by *in vitro* re-isolations.

The wood symptomatology caused by *P*. *chlamydospora*, when interacting with *E*. *layuense* E24, was consistently lower in both grapevine cultivars, with a 67.5% reduction of the brown wood streaking length in Cabernet Sauvignon and 73.8% in Touriga Nacional. Also for *P*. *minimum*, a significant reduction of symptoms was observed, although unevenly between cultivars (82% in Cabernet Sauvignon and 31.3% in Touriga Nacional) (Fig 9). Studies that described a similar reduction in the streaking length induced by *P*. *chlamydospora*, achieved with other antagonists, include *Pythium oligandrum* inoculated in the root system (40–50%) [16], *Trichoderma* spp. in nurseries and in pruning wound protection (67–79%) [20,69], *Bacillus pumilus* and *Paenibacullus* sp. (31–39%) in co-inoculation assays [14].

The frequencies of re-isolation of *P*. *chlamydospora* and *P*. *minimum*, when alone or interacting with *E*. *layuense*, support the observations gained from the measurements of the brown wood streaking. In fact, the shorter the streaking the lower the frequency of re-isolation of the pathogens (Figs 7 and 9). The examination of the wood at different distances from the pathogens inoculation point increased the understanding of their wood colonization success. While the pathogens presence is similar to that of the positive controls near the inoculation point (15mm below), it is considerably reduced further down the wood (45mm) (Fig 9). This suggests that the presence of *E*. *layuense* E24 slowed-down or completely stopped the spread of the pathogens in the wood. These observations also confirm the general understanding that the brown wood streaking length, although not incited exclusively by *P*. *chlamydospora* and *P*. *minimum*, is linked to the re-isolation of these pathogens [7,70].

Combining the length of brown wood streaking with the re-isolation of pathogens, it is interesting to notice how fast *P*. *minimum* and *P*. *chlamydospora* could colonize woody tissues, approximately 10 mm/month for the first and 17 mm/month for the second, under our experimental conditions. These data are particularly worrying when thinking about nursery infections, as pathogens may fully colonize newly rooted cuttings in a matter of months.

The application of biological control agents in perennial plants presents several challenges and its success can be influenced by biotic and abiotic factors. This study examined the potential of *Epicoccum layuense* E24 in the biological control of two esca-associated pathogens, under greenhouse conditions. Further investigation is required to assess whether the success of the wood colonization by *E*. *layuense* E24 and the antagonism observed vary in time (e.g. aging of the vine) and under field conditions (e.g. seasonality, extreme weather conditions). Infections of newly grafted rooted cuttings or young grapevines have been widely reported [10,71–74]; they entail weaker and less productive plants, leading often to death of the infected vines. From our results, it can be hypothesized that the application of *E*. *layuense* E24, or even its metabolites, in nurseries or young vineyards may help the young plants to cope with early infections, by reducing both the wood colonization by *P*. *chlamydospora* and *P*. *minimum* as well as the brown streaking symptom associated with them. The modes of production, delivery and application of *E*. *layuense* E24 need to be further investigated. In conclusion, *E*. *layuense* E24 is a promising candidate for biological control and its future application in nurseries and young vineyards is the natural follow-up of this study, in the ultimate quest of keeping in check these esca-related pathogens.

## Supporting information

S1 Fig Dual culture, in Petri dishes, of *Phaeomoniella chlamydospora* and *Epicoccum layuense* E24, 5 (a), 8 (b), 10 (c), 12 (d) and 14 (e) days post-inoculation. Scale bar = 10 mm.
(TIF)

S2 Fig Dual culture, in Petri dishes, of *Phaeoacremonium minimum* and *Epicoccum layuense* E24, 5 (a), 8 (b), 10 (c), 12 (d) and 14 (e) days post-inoculation. Scale bar = 10 mm.
(TIF)

S3 Fig Dual culture, in Petri dishes, of *Fomitiporia mediterranea* and *Epicoccum layuense* E24, 5 (a), 8 (b), 10 (c), 12 (d) and 14 (e) days post-inoculation. Scale bar = 10 mm.
(TIF)
