# Supplementary figures and images for: Epicoccum layuense a potential biological control agent of esca-associated fungi in grapevine
Source: PLoS One. 2019 Mar 26;14(3):e0213273. doi: 10.1371/journal.pone.0213273 (PMC6435229; doi:10.1371/journal.pone.0213273)

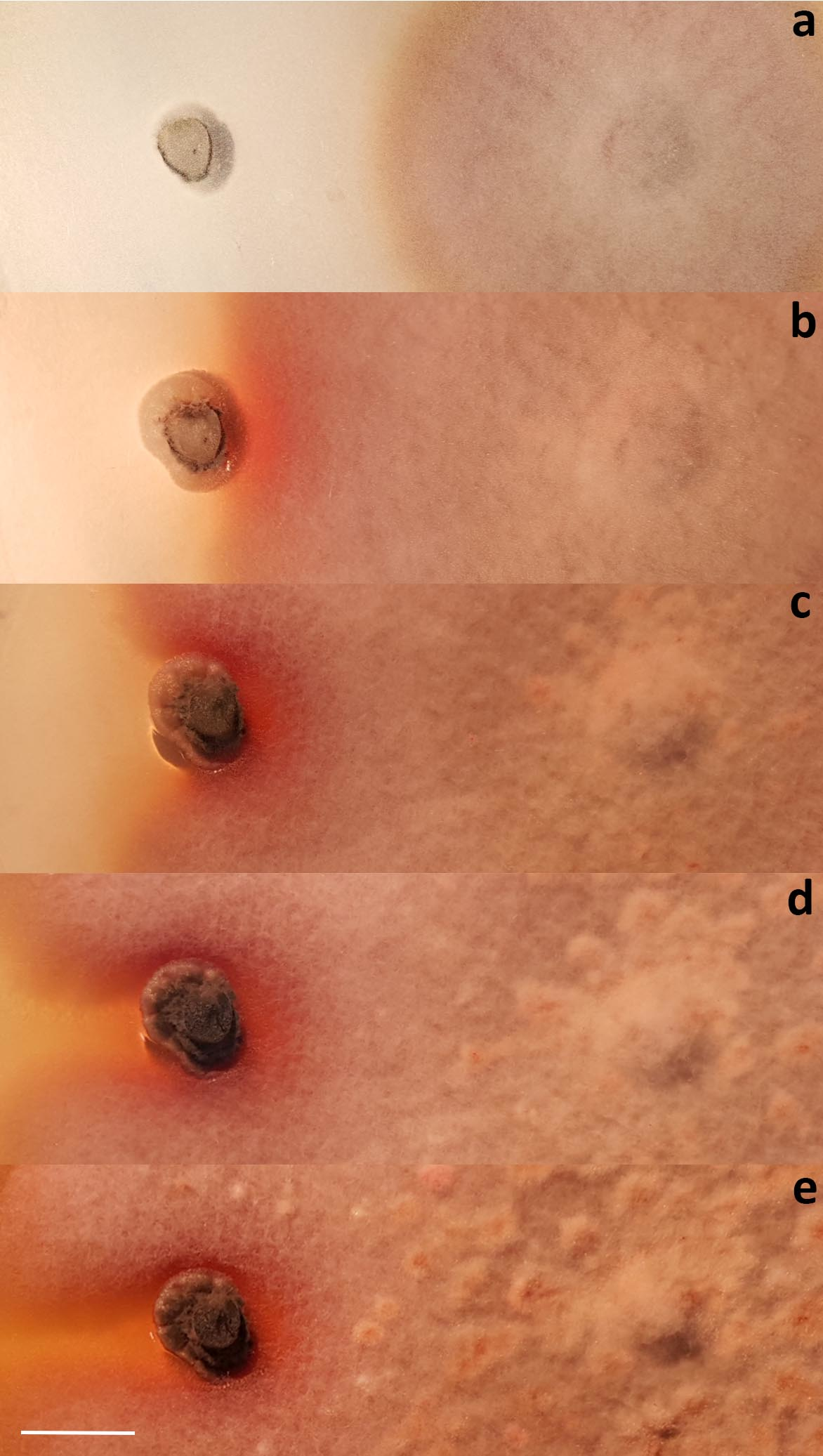

Supplement: S1 Fig — Scale bar = 10 mm. (TIF) [file pone.0213273.s001.tif]

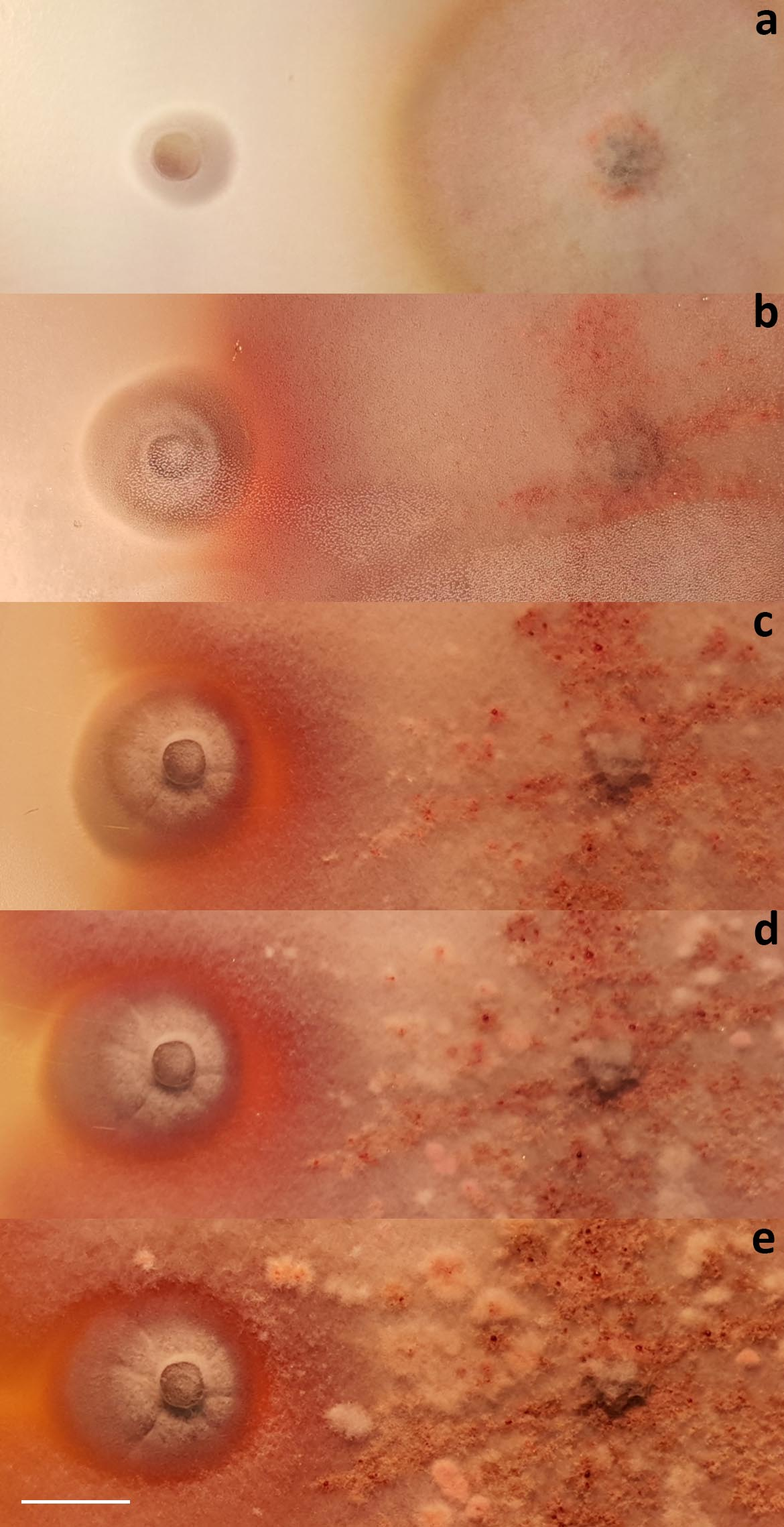

Supplement: S2 Fig — Scale bar = 10 mm. (TIF) [file pone.0213273.s002.tif]

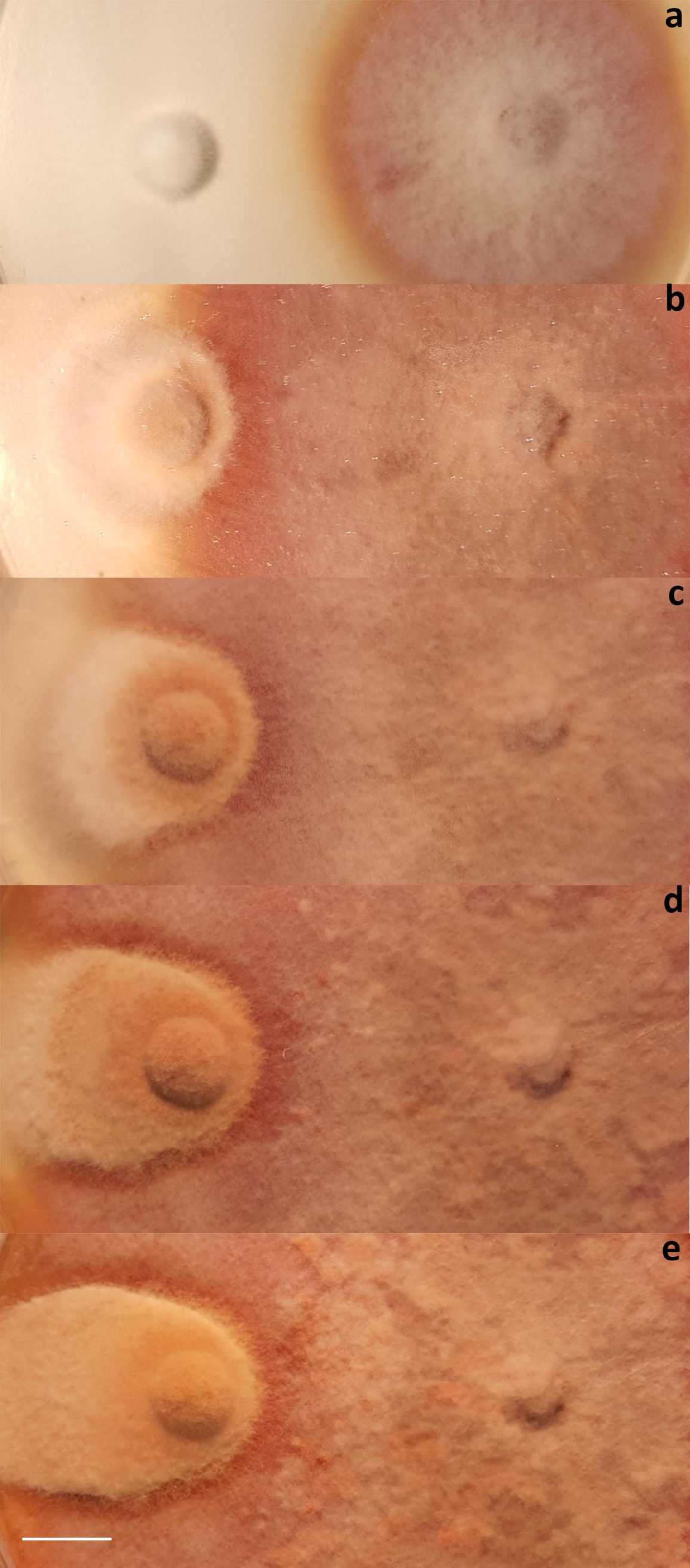

Supplement: S3 Fig — Scale bar = 10 mm. (TIF) [file pone.0213273.s003.tif]
